# Xpert MTB/RIF ultra for rapid diagnosis of extrapulmonary tuberculosis in a high-income low-tuberculosis prevalence setting

**DOI:** 10.1038/s41598-020-70613-x

**Published:** 2020-08-18

**Authors:** Ida Marie Hoel, Heidi Syre, Ingerid Skarstein, Tehmina Mustafa

**Affiliations:** 1 Department of Global Public Health and Primary Care, Centre for International Health, University of Bergen, Bergen, Norway; 2 Department of Medical Microbiology, Stavanger University Hospital, Stavanger, Norway; 3 Department of Microbiology, Haukeland University Hospital, Bergen, Norway; 4 Department of Thoracic Medicine, Haukeland University Hospital, Bergen, Norway

**Keywords:** Tuberculosis, Infectious-disease diagnostics

## Abstract

The diagnosis of extrapulmonary tuberculosis (EPTB) is often challenging due to paucibacillary nature of the disease. Xpert MTB/RIF Ultra (Ultra) has been developed to improve detection of *Mycobacterium tuberculosis* complex (MTC) in paucibacillary specimens. The objective of the study was to assess the performance of Ultra for the diagnosis of EPTB in a high-income low TB prevalence country. Extrapulmonary samples received for TB diagnostics at two hospitals in Norway between January 2015 and January 2016 were prospectively and consecutively included. Defrosted samples were subjected to Ultra. Culture and routine PCR tests were used as reference standard. A total of 82 samples, 10 culture and/or routine PCR positive (confirmed TB) samples and 72 culture and routine PCR negative samples were included in analysis. The overall sensitivity and specificity of Ultra were 90% (9/10, 95% CI 56–100) and 99% (71/72, 95% CI 93–100), respectively. Ultra was positive in 6/7 smear negative confirmed TB samples. To conclude, Ultra showed a high sensitivity and specificity in extrapulmonary specimens and may contribute to a rapid diagnosis of EPTB in a low TB prevalence setting.

## Introduction

Tuberculosis (TB) is a global health problem^1^. Extrapulmonary TB (EPTB) accounts for approximately 15% of notified TB cases globally^1^, whereas as much as 40% of TB cases are extrapulmonary in several high-income countries, including Norway^1,2^. Due to paucibacillary nature of the disease, the diagnosis of EPTB is often challenging. The worldwide roll-out in 2010 of the new PCR-based assay, Xpert MTB/RIF (Xpert; Cepheid, Sunnyvale, CA), represented a breakthrough in TB diagnostics^3^. The rapid and fully automated assay simultaneously detects *Mycobacterium tuberculosis* complex (MTC) species, the causative agents of TB, and rifampicin resistance (RIF-R), and has a high sensitivity for diagnosing pulmonary TB (PTB) in smear positive sputum samples^4^. However, the sensitivity of Xpert in paucibacillary specimens, including smear negative PTB and many forms of EPTB, is limited^4–6^. To improve the performance of Xpert in smear negative samples, an upgraded version of the assay, Xpert MTB/RIF Ultra (Ultra), has been developed^7^. Ultra was launched in 2017 and is recommended by the World Health Organization as a replacement for Xpert in all settings^8^. The increased sensitivity of Ultra is achieved by incorporation of two new PCR assays targeting the multicopy genes *IS6110* and *IS1081* for the diagnosis of TB, a larger DNA reaction chamber and transformation from hemi-nested to fully nested PCR reactions^7^. A number of studies report increased sensitivity of Ultra compared to Xpert in smear negative PTB^9–16^, and several studies also show promising results for diagnosing EPTB^11,14,15,17–25^. However, most of the studies that investigate Ultra for diagnosing EPTB have been conducted in low-resource settings with high TB incidence. The aims of the present study were to (1) evaluate the diagnostic accuracy of Ultra for diagnosing EPTB compared to routinely used culture and PCR tests in a clinical setting in the high-income low TB prevalence country Norway^26^, and (2) investigate the potential of Ultra as an add-on test to the existing routine tests to improve the rapid diagnosis of EPTB.

## Methods

### Sample inclusion

The present study was performed on frozen specimens that had been collected as part of a larger prospective study conducted at Haukeland University Hospital, Bergen, Norway, between January 2015 and June 2016^27^. The study includes 47 specimens that were also included in the larger study, and 39 additional specimens that did not meet the inclusion criteria in the larger study. The eligibility criteria and reference standard were designed prior to Ultra testing. Samples eligible for inclusion were identified at the microbiology laboratories at two regional tertiary care hospitals, Haukeland University hospital and Stavanger University hospital. All consecutive extrapulmonary samples received for TB diagnostics from patients of all ages were included during the study period, provided there was enough material left after routine diagnostics. As the diagnosis of EPTB almost always requires invasive sample collection, which is only performed on symptomatic patients with abnormal tissue masses or fluids, we assumed that the pre-test probability of TB was generally quite high in these samples. One exception was pleural fluid samples, which accounted for a large proportion of the samples, but often had a very low pre-test probability for TB because most of the samples were malignant pleural effusions routinely sent for TB diagnostics before initiation of cancer chemotherapy. Hence, pleural fluid samples were only included and subjected to Ultra if TB was mentioned as a probable differential diagnosis on the request form, whereas all other sample types were included without selection. Multiple samples were taken from some patients, and individual patients were allowed to contribute to the dataset multiple times. Results of routine TB diagnostic tests were obtained from the microbiology laboratory information systems. A microbiological reference standard was used in this study. Culture and/or routine PCR test positive samples were categorised as confirmed TB samples, and culture and routine PCR test negative samples were categorised as non-TB samples. Because information about clinical TB diagnosis was not available for all the samples in the cohort, we could not include a clinical TB diagnosis as part of the reference standard, and any sample included from a clinically diagnosed TB case was therefore categorised as a non-TB sample in this study.

### Sample processing and routine TB diagnostic procedures

Laboratory personnel at the inclusion hospitals performed routine TB diagnostics according to local diagnostic algorithms. They were blinded to Ultra results. Fine needle aspirates (FNAs) and fluid samples with a volume of < 10 mL were used unconcentrated, whereas samples with a volume of > 10 mL were concentrated by centrifugation (3,800×*g* for 15 min) before resuspension of the sediment in saline. Biopsy specimens were mechanically homogenized and resuspended in saline. The Ziehl–Neelsen (ZN) method was used for detection of acid fast bacilli by smear microscopy. At Haukeland University hospital, a standard NALC-NaOH decontamination procedure was performed if the sample was assumed to be non-sterile, before seeding of appropriate sample volumes in liquid medium (BACTEC MGIT) and solid medium (Lowenstein-Jensen containing glycerol and sodium pyruvate). All lymph node specimens, sterile fluids and aspirates and most biopsies were cultured both before and after NALC-NaOH decontamination, and lymph node specimens were also cultured at 28 °C. At Stavanger University hospital, most extrapulmonary samples were NALC-NaOH decontaminated and only cultured on liquid medium (Bactec MGIT 960; Becton Dickinson, Towson, MD). If the clinician requested PCR, appropriate sample volumes were further used for Cobas Taqman MTB (Roche, Switzerland), Abbott Real Time MTB (Abbot, Des Plaines, IL) or Genotype MTBDR plus (Hain Lifescience, Nehren, Germany), hereafter collectively referred to as routine PCR. Any remaining sample material was stored at − 80 °C for later analysis with Ultra.

### Xpert ultra

We performed Xpert ultra on the frozen sample material during the autumn 2018, blinded for results of routine TB diagnostics and clinical information. Samples were thawed at room temperature and processed according to the manufacturer’s protocol. All but two samples (both volume 0.25 mL) had a sample volume of minimum 0.5 mL. In samples with volume < 0.7 mL (n = 32), sample reagent was added in a 3:1 reagent to sample ratio, whereas a ratio of 2:1 was used for samples with a volume of 0.7 mL or more (n = 54).

### Statistical analysis

Sensitivity, specificity and accuracy were calculated using culture and routine PCR as the reference standard. A minimum of one valid (positive or negative) culture result and a valid Ultra result were required to include a sample in this analysis. Calculation of 95% confidence intervals for sensitivity and specificity was performed with the exact Clopper-Pearson method.

### Ethical considerations

The study was approved by the Regional Committee for Medical and Health Research Ethics of Western Norway (REK Vest), (2014/46/REK vest), and carried out in accordance with relevant guidelines and regulations. REK Vest granted an exemption from informed consent from the patients, as the study only included residual material from samples sent for TB diagnostics in a clinical setting.

## Results

**Figure 1:**
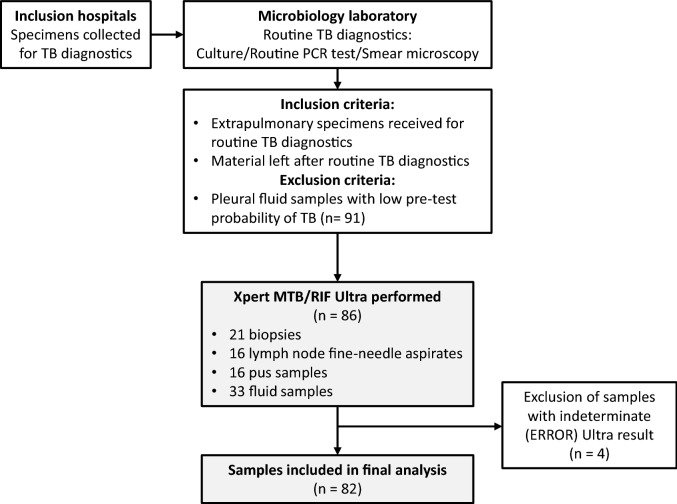
Overview of study design. The type of routine PCR test performed varied between the inclusion hospitals, and included Cobas Taqman MTB (Roche, Switzerland), Abbott Real Time MTB (Abbot, Des Plaines, IL) and Genotype MTBDR plus (Hain Lifescience, Nehren, Germany). *TB* tuberculosis, *PCR* polymerase chain reaction.

Figure 1 provides the study overview. Frozen material was available from a total of 177 samples received for TB diagnostics during the study period, and comprised 21 biopsies, 16 lymph node FNAs, 16 pus samples and 124 fluid samples. Pleural fluid samples accounted for more than half of all the specimens (n = 109), but TB was only mentioned as a probable differential diagnosis on the request form for 18/109 samples. We excluded the 91 pleural fluids with assumed low pre-test probability of TB and analysed the remaining 86 extrapulmonary samples (from 80 cases) with Ultra. All these samples had a minimum of one valid culture result (positive or negative) available. Four samples were excluded from further analysis due to invalid Ultra result (ERROR).

The type and number of routine TB diagnostic tests performed on the analysed samples varied (Table 1). All samples were subjected to culture, 85% to ZN microscopy (n = 70) and 24% to a routine PCR (n = 20). MTC was detected in ten samples (positive culture and/or routine PCR), hereafter called confirmed TB samples, while 72 samples were culture and routine PCR negative (Table 1). HIV status was unknown for all cases.

**Table 1:** Distribution of routine TB diagnostic tests performed and results of routine TB diagnostic tests and Xpert MTB/RIF Ultra.

Culture and/or PCR positive samples^a^	Number of samples	Routine TB diagnostic tests (positive/total number)			Xpert Ultra
		Microscopy	Routine PCR	Culture	
Lymph node biopsy	1	0/1	1/1	1/1	1/1
Lymph node aspirate	4	3/4	3/3	4/4	4/4
Pus samples	2	0/2	2/2	1/2	2/2
Other biopsies (pleura)	1	0/1	0/1	1/1	1/1
Fluid samples	1	0/1	0/1	1/1	0/1
Gastrointestinal lavage	1	0/1	1/1	1/1	1/1
Total	10	3/10	7/9	9/10	9/10
**Samples per inclusion site**					
Samples from HUH	8	3/8	5/7	8/8	7/8
Samples from SUH	2	0/2	2/2	1/2	2/2
**Culture and PCR negative samples**^b^					
Lymph node biopsy	4	1/4	0/1	0/4	1/4
Lymph node aspirate	11	2/11^c^	0/3	0/11	0/11
Pus samples	14	1/14^d^	0/1	0/14	0/14
Other biopsies	13	0/13	0/4^e^	0/13	0/13
Fluid samples	28	0/17	0/3	0/28	0/28
Gastrointestinal lavage	2	0/1	N/A	0/2	0/2
Total	72	4/60	0/12	0/72	1/72
**Samples per inclusion site**					
Samples from HUH	58	3/53	0/9	0/58	0/58
Samples from SUH	14	1/7	0/3	0/14	1/14

*HUH* Haukeland University Hospital, *SUH* Stavanger University Hospital.

^a^Two of the samples (culture positive biopsy and culture positive fluid sample) are from the same TB case.

^b^Includes three samples from clinically diagnosed TB cases (one pus sample, one biopsy and one lymph node aspirate).

^c^The two microscopy positive samples were both culture positive for *Mycobacterium avium.*

^d^Microscopy positive sample was culture positive for *Mycobacterium avium.*

^e^Five culture negative biopsy samples were subjected to routine PCR, of which one PCR test result was indeterminate (technical failure). The indeterminate PCR result has been excluded from the results and analysis.

### Test performance of ultra compared to routine TB diagnostic tests

**Figure 2:**
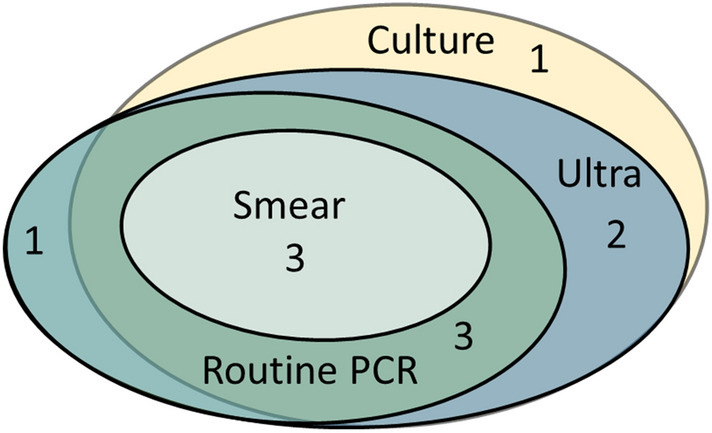
Euler diagram illustrating overlap of positive results of routine TB diagnostic tests and Xpert MTB/RIF Ultra in confirmed TB samples (n = 10).

Using culture and/or routine PCR as reference standard, Ultra was positive in 9 of 10 confirmed TB samples, giving an overall sensitivity of 90% (95% CI 56–100) (Table 2). Among the nine culture positive TB samples, Ultra was positive in eight (Fig. 2). All the smear positive (3/3) and 6/7 (86%) smear negative confirmed TB samples were Ultra positive. Semi-quantitation of bacillary load by Ultra categorised the TB samples as medium (n = 3), low (n = 3), very low (n = 1) and trace (n = 2). None of the TB samples had a high bacillary load. Among the 8 culture and Ultra positive samples, genotypic RIF-R was detected by Ultra in two samples, not detected in four samples and indeterminate in the two samples semi-quantitated as “trace”. The genotypic RIF-R results were in concordance with the phenotypic drug susceptibility test (DST) results. The two samples with indeterminate RIF-R by Ultra were both sensitive to first line TB drugs in phenotypic DST.

**Table 2:** Validation of Xpert MTB/RIF Ultra using culture and/or routine PCR tests as a reference standard.

Sample material	Sensitivity	Specificity	PPV	NPV	Accuracy
	% (95% CI) TP/(TP + FN)	% (95% CI) TN/(TN + FP)	% TP/(TP + FP)	% TN/(TN + FN)	% (95% CI) (TP + TN)/(TP + FP + FN + FP)
All samples	90 (56–100) 9/10	99 (93–100) 71/72	90 9/10	99 71/72	98 (91–100) 80/82
Lymph node biopsy	100 (3–100) 1/1	75 (19–99) 3/4	50 1/2	100 3/3	83 (6–100) 4/5
Lymph node aspirate	100 (40–100) 4/4	100 (72–100) 11/11	100 4/4	100 11/11	100 (78–100) 15/15
Pus samples	100 (16–100) 2/2	100 (77–100) 14/14	100 2/2	100 14/14	100 (79–100) 16/16
Other biopsies	100 (3–100) 1/1	100 (75–100) 13/13	100 1/1	100 13/13	100 (77–100) 14/14
Fluid samples	0 (0–98) 0/1	100 (88–100) 28/28	0 N/A	97 28/29	97 (82–100) 28/29
Gastrointestinal lavage	100 (3–100) 1/1	100 (16–100) 2/2	100 1/1	100 2/2	100 (30–100) 3/3

95% confidence intervals for sensitivity, specificity and accuracy were calculated using the exact Clopper–Pearson method.

*CI* confidence interval, *TP* true positive, *FN* false negative, *TN* true negative, *FP* false positive.

One of the 72 culture and routine PCR negative samples was Ultra positive, yielding an overall specificity of 99% (95% CI 93–100) for Ultra. This sample was a lymph node biopsy from a patient with lymphadenitis following BCG vaccination. The lymphadenitis healed spontaneously and was eventually interpreted as an immune reaction to the vaccine. Among the culture and routine PCR negative samples were also three non-tuberculous mycobacteria (NTM) culture positive samples, identified as *Mycobacterium avium*. All three were Ultra negative.

## Discussion

In the present study we have investigated the performance of Ultra compared to routine TB diagnostic tests for diagnosing EPTB in a small cohort of prospectively collected extrapulmonary specimens in a high resource setting with a low TB prevalence. Using culture and/or routine PCR as reference standard, we found that Ultra had an overall sensitivity and specificity of 90% (95% CI 56–100) and 99% (95% CI 93–100), respectively. In smear negative confirmed TB samples, the sensitivity of Ultra was 86% (95% CI 42–100).

Several studies have been published on the diagnostic accuracy of Ultra in extrapulmonary specimens^11,14,15,17–25,28,29^. Most of the studies were performed in TB endemic settings^14,17,19–25,28,29^, and only three studies have investigated Ultra test performance in low TB prevalence settings^11,15,18^. However, two of these studies were retrospective and performed on selected sample material. A strength of our study is that it is a prospective cohort study with consecutive inclusion of samples in a clinical routine setting, and the study population is thus more likely to be representative of the true test population in a low TB prevalence setting. The majority of extrapulmonary samples tested for TB in Norway are from patients with other diseases than TB. Hence, it may be just as important to avoid false positive test results in the large group of non-TB cases, which can lead to overtreatment and potentially severe side effects, as to obtain a more rapid TB diagnosis in the small group of TB cases. Our study can provide useful information about test performance in these clinically relevant non-TB cases, which we think is of particular importance in our setting. Indeed, the specificity of Ultra in our study was high (99%). This is in concordance with the specificities (97–100%) found in other low TB prevalent settings where culture was used as reference standard^15,18^. However, in most of the studies that directly compare the performance of Xpert and Ultra, the specificity of Ultra is reduced compared to Xpert, both in extrapulmonary specimens^14,19,20,22,29^ and sputum^9,10,12,13^. In low TB prevalent settings, even a small reduction in specificity could lead to unacceptable high rates of false positive cases, emphasizing that Ultra should be performed on selected samples with a high pre-test probability of TB disease.

Only one culture and routine PCR negative sample in our cohort was Ultra positive (“trace”). The sample came from a patient with regional lymphadenitis following BCG vaccination. As the BCG strains are also members of the MTC^30,31^, the Ultra result may have been true positive for this case. However, Ultra is not able to separate non-viable from viable bacilli and cannot provide information as to whether the DNA present in the sample represented active mycobacterial disease or remnants of old DNA from the vaccine. This illustrates the challenges in interpreting positive Ultra results in certain clinical contexts. The inability of Ultra to separate viable from non-viable bacilli also limits its use for detection of relapse and reinfection. Dorman and colleagues reported a reduced specificity of Ultra in previously treated TB patients up to seven years after completion of treatment compared to patients with no previous history of TB^9^. This underlines the importance of culture for TB diagnosis.

Most, but not all^28,29^, of the studies that investigate Ultra for the diagnosis of EPTB suggest increased sensitivity of Ultra compared to Xpert in different extrapulmonary specimens^11,14,15,17,19–23,25^. When culture is used as reference standard, the reported sensitivity of Ultra varies, but is generally high in lymph node specimens (90–94%)^18,24^, FNAs and tissue samples (87–95%)^18,22^, cerebrospinal fluid (80–100%)^17,18,21,25,29^ and pus specimens (65–95%)^18,19^, and lower in pleural fluid samples (48–84%)^18,20,22,23^, which is in line with our findings. Several of the studies also show that Ultra can detect MTC in culture negative specimens from clinically diagnosed TB cases^17,19–25,28,29^, thus, contributing to improved diagnosis of TB in samples with a very low bacillary load.

The ability of Xpert and Ultra to simultaneously detect MTC and RIF-R is considered one of the strengths of the assays. In addition to detection of the single-copy *rpoB* gene for the simultaneous diagnosis of TB and rifampicin resistance, Ultra includes two new and more sensitive PCR assays that target the multicopy *IS6110* and *IS1081* genes to improve MTC detection in paucibacillary samples^7^. However, the increased sensitivity of the test comes with an expense, because information about RIF-R is not available in *IS6110 or IS1081* positive and *rpoB* negative samples, which are categorised as “trace” by Ultra^7,18^. Two of the culture confirmed TB samples in our study were Ultra “trace” positive. For these samples, information about drug resistance was only provided by culture, and both were drug sensitive. The incidence of multidrug resistant (MDR)-TB is low in our setting^26^, but in high MDR-TB incidence settings, MDR-TB cases can be missed among the “trace” positive samples. Therefore, Ultra should complement, but not replace culture.

This study has some limitations. The small sample number leads to uncertain sensitivity estimates. The study was performed in a clinical routine setting, and the combination of routine TB diagnostics used and sample processing varied slightly between the inclusion hospitals. The number of cultures performed per sample and the use of decontaminated versus not-decontaminated material may affect the sensitivity of culture. At both inclusion sites, fluid samples and FNAs with a volume of > 10 mL were concentrated before use. Hence, we cannot evaluate the overall effect of sample concentration on Ultra test performance, as some samples were concentrated and others were not. The use of frozen sample material for Ultra may also affect the performance of the assay due to reduced sample quality. However, studies that have specifically investigated the Xpert/Ultra test performance on frozen samples report discordant results, but the effect is small in the studies that find a difference^6,12,18,32^. Furthermore, DNA remains quite stable under frozen conditions, and our samples were only subjected to one freeze–thaw cycle. These findings imply that the impact of frozen samples on Ultra performance is small. Larger and more controlled studies with uniform sample processing should be conducted to adjust for these limitations. As culture is known to be an imperfect reference standard in paucibacillary cases of EPTB, future studies should focus on diagnostic accuracy of TB diagnostic tests both in microbiologically confirmed and clinically diagnosed TB subjects to better reflect the true test performance in all clinically relevant groups of the test population.

To conclude, the sensitivity and specificity of Ultra was comparable to culture in most sample materials in this study with the advantage of results within hours. These promising findings suggest that Ultra is a useful add-on test that can contribute to a rapid diagnosis of EPTB in our setting. However, the inability of Ultra to separate viable from non-viable bacilli and the lack of information about drug resistance for “trace” positive samples, limits its use in some clinical cases, especially in patients previously treated for TB.

## Data Availability

The datasets generated and analysed during the current study are available from the corresponding author on reasonable request.
